# Supplementary material for: Multidirectional digital scanned light-sheet microscopy enables uniform fluorescence excitation and contrast-enhanced imaging
Source: Sci Rep. 2018 Sep 17;8:13878. doi: 10.1038/s41598-018-32367-5 (PMC6141597; doi:10.1038/s41598-018-32367-5)
Supplement: Supplementary file 1 — Supplementary Material [file 41598_2018_32367_MOESM1_ESM.docx]

**Title:** Multidirectional digital scanned light-sheet microscopy enables uniform fluorescence excitation and contrast-enhanced imaging

**Authors:** Adam K. Glaser^1*^, Ye Chen^1^, Chengbo Yin^1^, Linpeng Wei^1^, Lindsey A. Barner^1^, Nicholas P. Reder^2^, and Jonathan T.C. Liu^1,2*^

**Affiliations:**

^1^Department of Mechanical Engineering, University of Washington, Seattle, WA USA.

^2^Department of Pathology, University of Washington, Seattle, WA USA.

*To whom correspondence should be addressed: [akglaser@uw.edu](mailto:akglaser@uw.edu) and [jonliu@uw.edu](file:///C:\Users\AERB\Downloads\jonliu@uw.edu).

**One Sentence Summary:** Glaser *et al.* describe a light-sheet microscopy architecture that enables passive multidirectional illumination with confocal line detection to enable both uniform fluorescence excitation and contrast-enhanced imaging of fluorescently labeled samples.

**Supplementary material**

**Text**2

Alternative mSPIM and mDSLM architectures 2

**Figures**3

SPIM, mSPIM, DSLM, and mDSLM imaging of a piece of fibrous human breast tissue3

Two color SPIM, mSPIM, DSLM, and mDSLM imaging of a piece of human breast tissue4

SPIM, mSPIM, DSLM, and mDSLM imaging of human breast tumor tissue5

Multidirectional architectures using a diffraction grating6

Multidirectional light sheet architecture using a transmission diffraction grating7

Multidirectional pencil beam architecture using a transmission diffraction grating8

Simulations and experimental measurements of *N* = 3 overlapping light sheets9

Experimental determination of the required scanning speed for dithering10

Experimental measurements of multidirectional illumination light sheet and pencil beams11

Experimental setup for the SPIM architecture12

Experimental setup for the mSPIM architecture13

Experimental setup for the DSLM architecture14

Experimental setup for the mDSLM architecture15

**Videos**16

Circular and elliptical Gaussian beams propagating through a glass sphere16

Comparison of SPIM, mSPIM, DSLM, and mDSLM imaging in biological tissue16

Video of *N* = 3 overlapping light sheets16

Video of *N* = 3 overlapping pencil beams16

**Alternative mSPIM and mDSLM architecture (diffraction-grating approach)**

Supplementary figures 4 – 9 describe an alternative method for the mitigation of shadowing artifacts, through the use of a diffraction grating to generate multiple angled light sheets (mSPIM) or Gaussian pencil beams (mDSLM) at discrete angles.

**Alternative mDSLM architecture (direct combination of DSLM and mSPIM methods)**

Another alternative to achieving the mDSLM principle is a direct combination of the mSPIM and DSLM concepts. Both a pivoting mirror and scanning mirror could be used in conjunction to pivot a circular Gaussian pencil beam rapidly at each beam scanning position, along with the use of a large confocal slit to avoid clipping the pencil beams too much as they pivot. However, this would require a high-speed pivoting mirror, as the pencil beam would need to pivot within the exposure time of the confocal slit, rather than the exposure time of the entire camera chip (as with mSPIM). Given the 50-Hz framerate of state-of-the-art sCMOS chips (with a rolling shutter), the pivoting mirror would need to scan as fast as ~200 kHz (assuming the beam pivots through one cycle within a 1 pixel confocal slit size). For a larger slit size such as the one used in this study ($\omega_{slit}$ = 20 μm or 25 pixels), the mirror would still need to pivot at ~8 kHz. While possible with current resonant scanners, a resonant scanner is more complex, expensive, and prone to failure than the completely passive approach of using a cylindrical telescope to generate an elliptical Gaussian beam. In addition, as sCMOS detector technologies improve in terms of speed, sensitivity, and functionality (e.g. the newest sCMOS cameras offer “dual” light-sheet readout modes where the framerate for confocal line imaging is increased to 100 Hz for two-color imaging), our passive multidirectional illumination approach does not impose additional speed constraints.

As mentioned previously, another downside of rotating a Gaussian pencil beam in a confocal DLSM system is that much of the beam would rotate out of a thin confocal slit (i.e. a confocal slit whose thickness, $\omega_{slit}$, is matched with the beam waist diameter). Therefore, in order to prevent a large loss of signal, a larger slit (larger rolling shutter) would likely be necessary. Note, also, that this loss of signal is worse than what would be expected with the static mDLSM approach, presented in the main manuscript, which utilizes an elliptical Gaussian beam. This is because with static mDLSM, the illumination pencil beam is always optimally aligned with the confocal slit (rolling shutter), in which the center of the Gaussian beam is never clipped by the slit. However, in a rotational mDLSM approach, much of the beam could rotate out of the confocal slit, including the center of the Gaussian beam (where most of the beam power is concentrated).


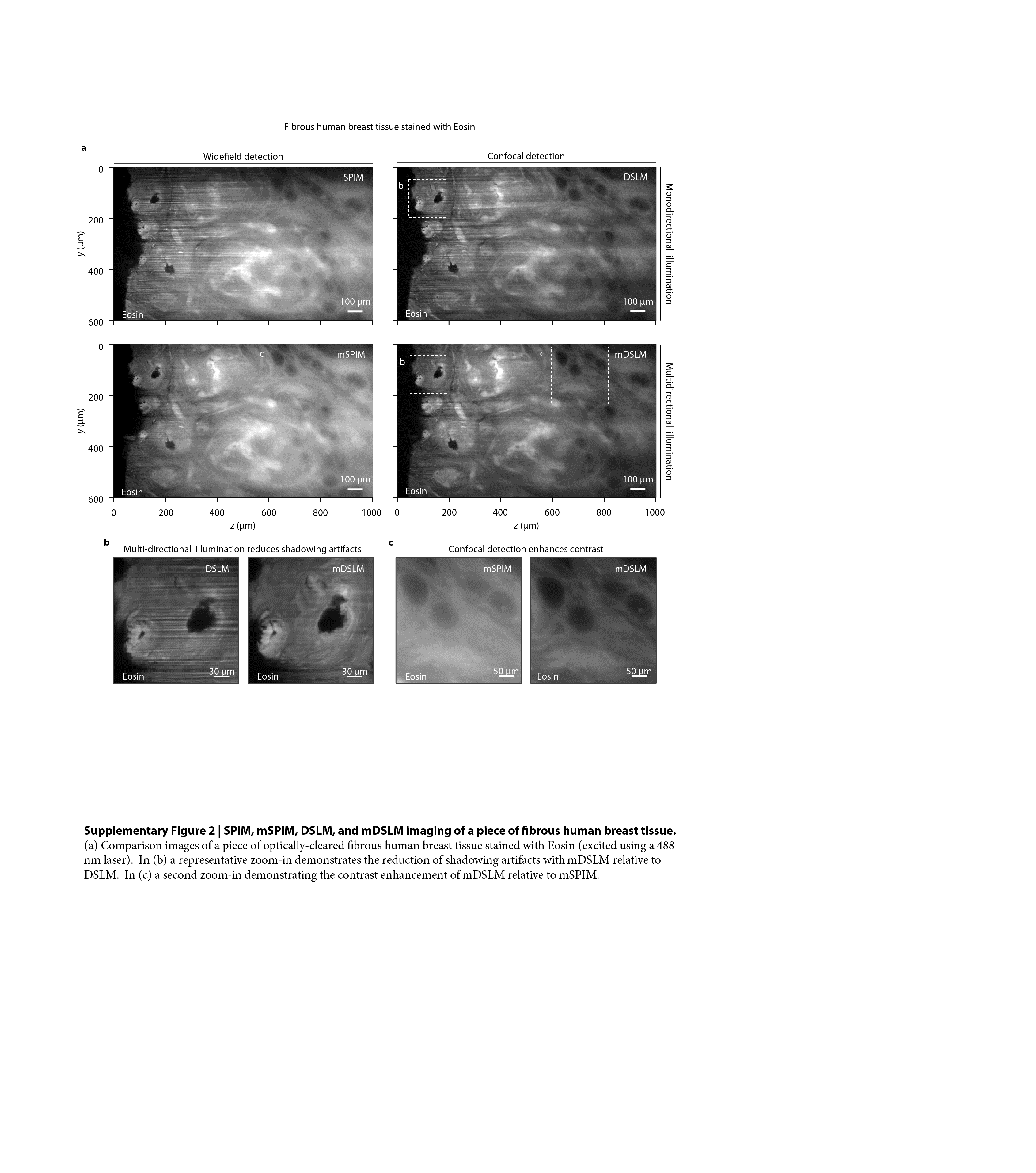


**Supplementary figure 1 | SPIM, mSPIM, DSLM, and mDSLM imaging of a piece of fibrous human breast tissue.** (a) Comparison images of a piece of optically-cleared fibrous human breast tissue stained with Eosin (excited using a 488 nm laser). In (b) a representative zoom-in demonstrates the reduction of shadowing artifacts with mDSLM relative to DSLM. In (c) a second zoom-in demonstrating the contrast enhancement of mDSLM relative to mSPIM.

**
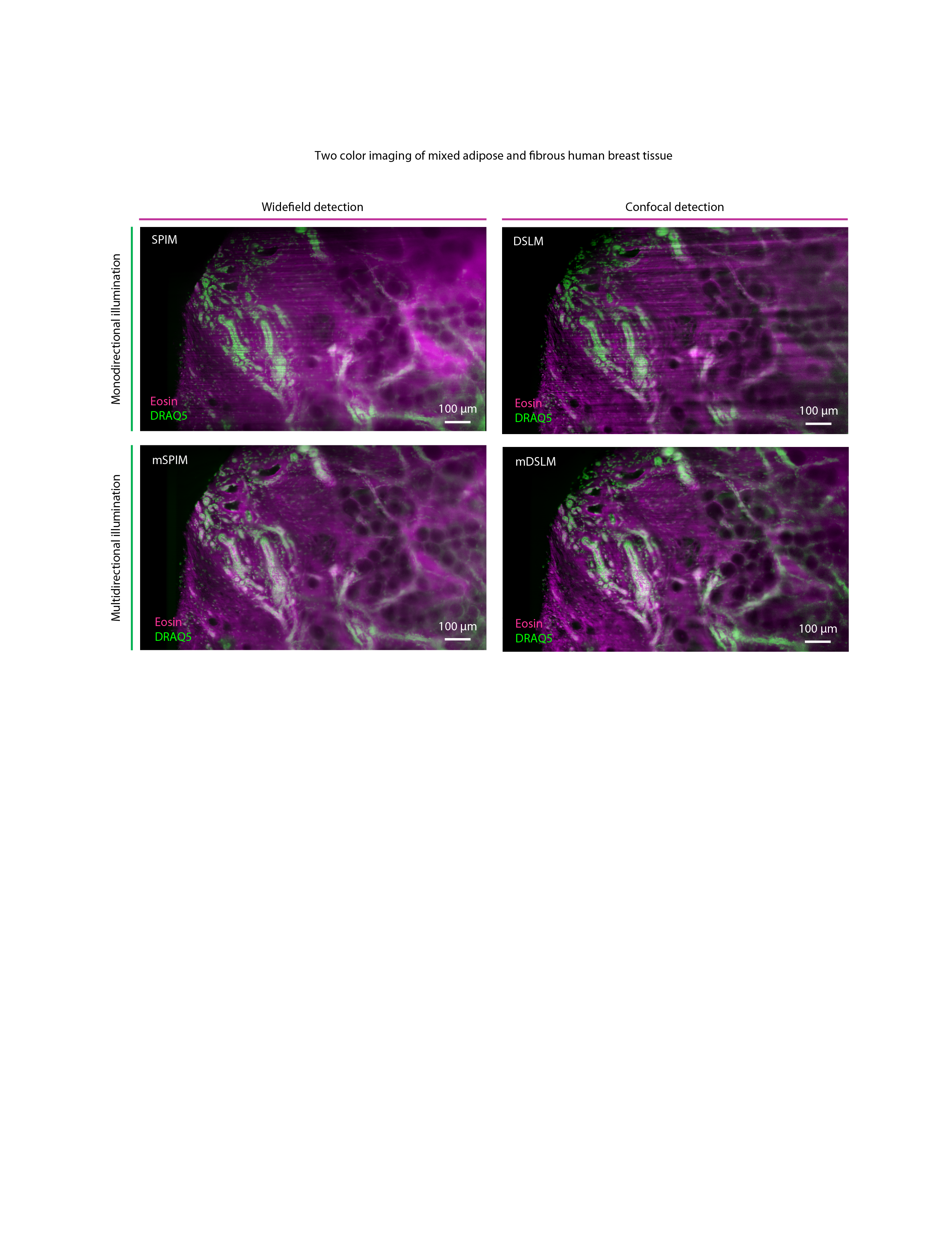
**

**Supplementary figure 2 | Two color SPIM, mSPIM, DSLM, and mDSLM imaging of a piece of human breast tissue.** (a) Comparison images of a piece of optically-cleared fibrous human breast tissue stained with Eosin (excited using a 488 nm laser, magenta) and DRAQ5 (excited using a 660 nm laser, green). The use of two colors demonstrates the compatibility of mDSLM with multicolor imaging.

**
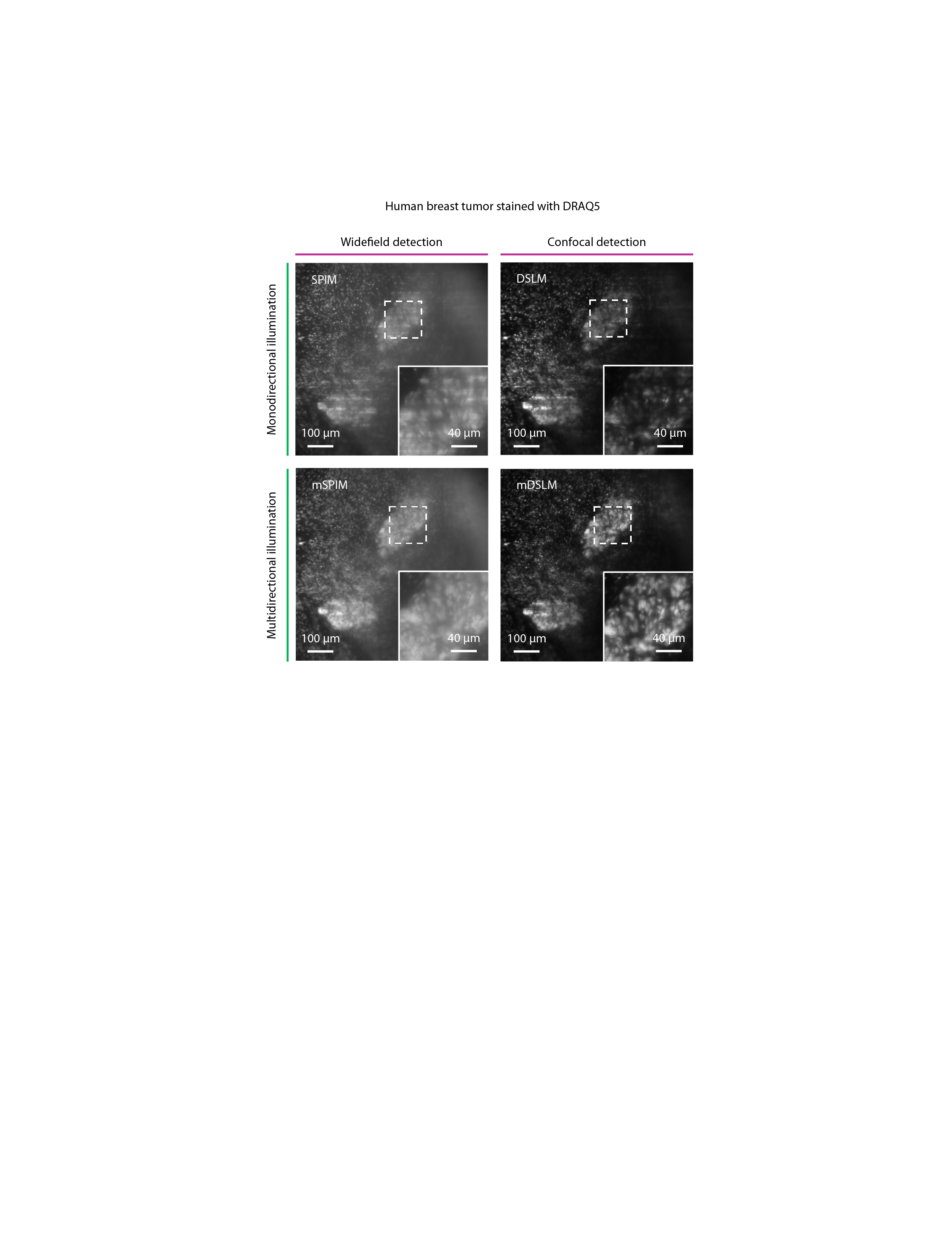
**

**Supplementary figure 3 | SPIM, mSPIM, DSLM, and mDSLM imaging of human breast tumor tissue**. Comparison images of an optically-cleared human breast tumor stained with DRAQ5 (excited using a 660 nm laser). Representative zoom-ins on a lobule containing dense nuclei demonstrates the shadow mitigation and contrast-enhancing ability of mDSLM.


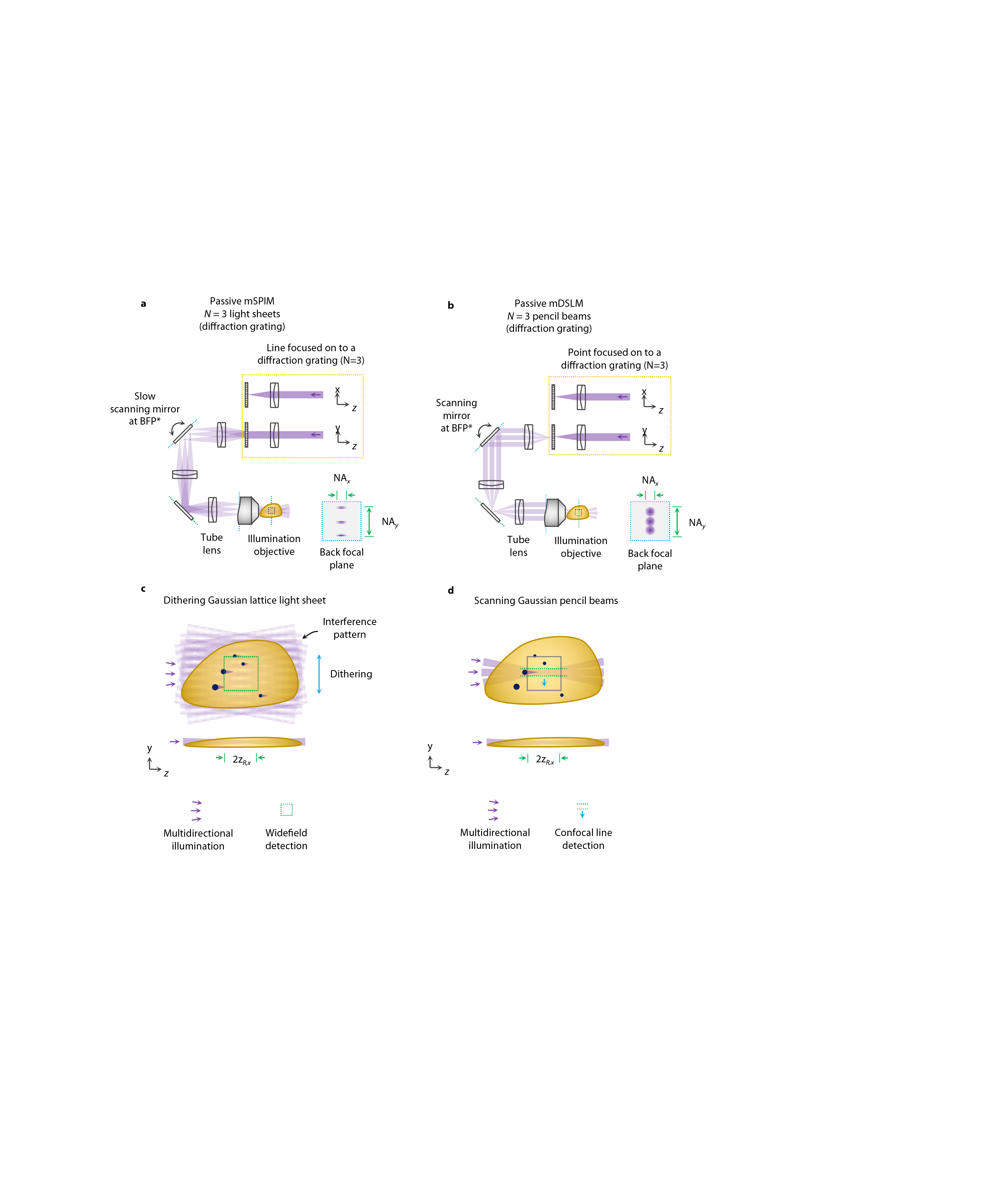


**Supplementary figure 4 | Multidirectional architectures using a diffraction grating.** (a) to mimic the mSPIM approach, a transmission diffraction grating is placed on a conjugate front focal plane of the illumination objective. A line is focused onto the grating (positioned at a conjugate front focal plane), which is dispersed into 3 diffraction orders (-1, 0, and 1), forming three line foci in the back focal plane of the illumination objective. The total separation between the -1 and +1 orders defines the effective span of NA*_y_*. The same concept for mDSLM is shown in (b), where a point is focused onto the transmission grating, resulting in three circular Gaussian beams in the back focal plane of the illumination objective. The centers of the -1 and +1 defines the effective span of NA*_y_*. In both cases, NA*_x_* is the same and remains unchanged by the one-dimensional diffraction grating. Zoom-in views of the illumination beams in the sample are shown in (c) and (d). In (c), the three tilted light sheets overlap, forming an interference pattern, or Gaussian lattice light sheet, due coherence. To form a uniform light sheet, the pattern must be dithered (relatively slowly) using a scanning mirror or pivoting mirror. This results in multidirectional illumination combined with widefield detection. In (d), the three tilted circular Gaussian beams overlap at the beam focus. The three beams are scanned in the *y*-direction and synced to the rolling shutter of the camera. This configuration results in multidirectional illumination combined with confocal line detection, analogous to mDSLM using an elliptical Gaussian beam. It should be noted that the use of a diffraction grating results in discrete low-NA beams oriented at multiple angles spanning NA*_y_*, as opposed to mSPIM and mDSLM that continuously fill the entire effective-NA*_y_* space. Also, although a transmission diffracting grating is used in these illustrations, a reflective diffraction grating (positioned at FFP*) could also be used to achieve the same illumination pattern within the sample.

**
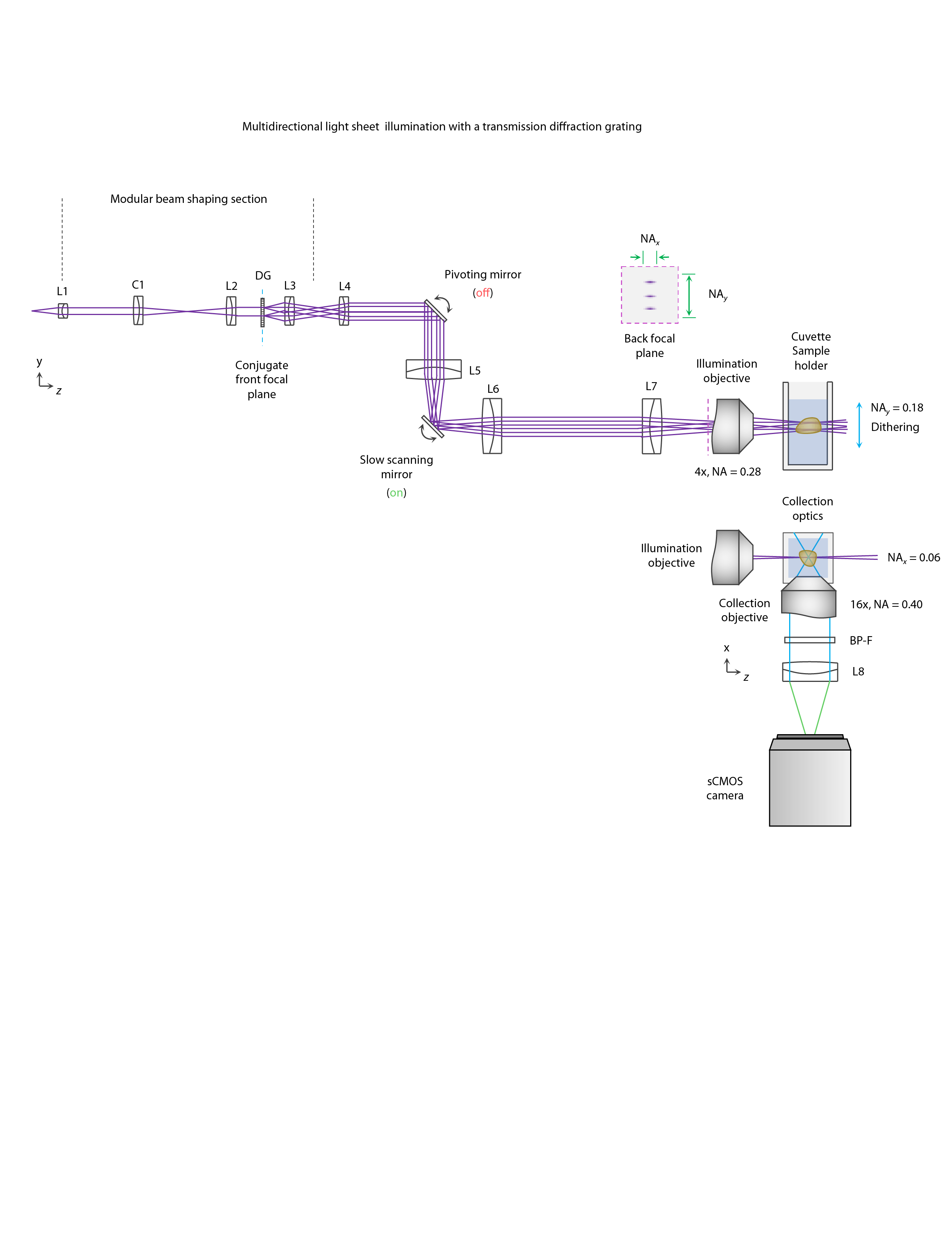
**

**Supplementary figure 5 | Multidirectional light-sheet architecture using a transmission diffraction grating.** Light enters the system using a 0.12 NA fiber-coupled laser and is collimated by lens L1 (*f* = 19 mm). The light is focused to a line by cylindrical lens C1 (*f* = 50 mm), and refocused by lens L2 (*f* = 50 mm) onto a transmission diffraction grating, DG (80 grooves/mm, Dynasil). The diffracted light (*N* = 3 orders at -10, 0, and +10 deg.) is imaged by lens L3 (*f* = 75 mm) and relayed by lens L4 (*f* = 200 mm) onto a pivoting mirror located in a conjugate front focal plane of the illumination objective. Light is collected by a scan lens, L5 (*f* = 70 mm), and imaged onto a second scanning mirror positioned in a conjugate back focal plane of the illumination objective. The scanned light is imaged by a second scan lens, L6 (*f* = 70 mm), and relayed to the back focal plane of the objective by a tube lens, L7 (*f* = 165 mm). Finally, the light is focused into the cuvette and sample by the illumination objective (4x, NA = 0.28), resulting in an effective illumination NA*_x_* = 0.06 and NA*_y_* = 0.18. Only the scanning mirror is on for the mSPIM architecture using a transmission diffraction grating. The excited fluorescence is detected by a collection objective (16x, NA = 0.40), filtered by a bandpass filter, BP-F, and imaged onto a sCMOS camera using a tube lens, L8 (*f* = 100 mm).

**
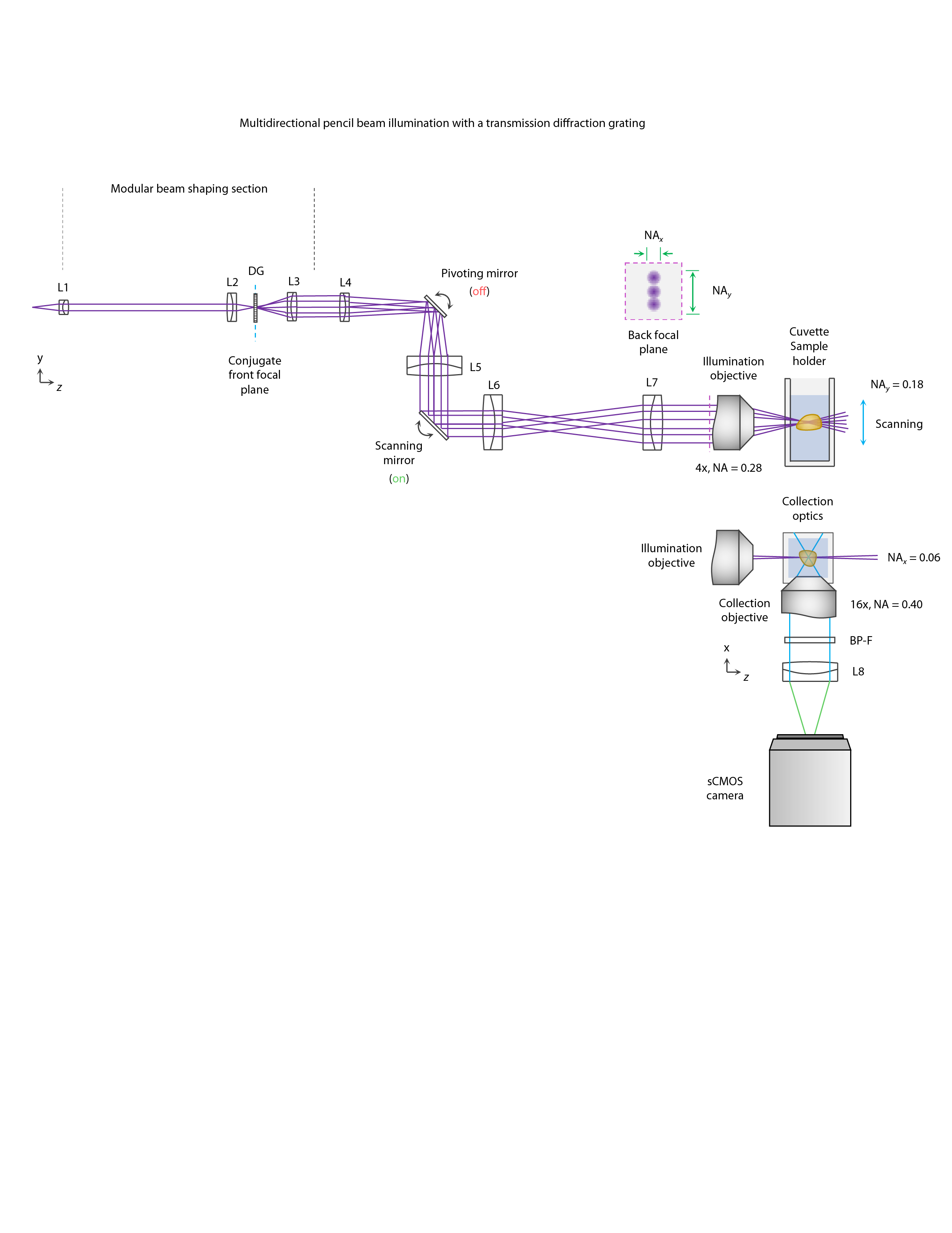
**

**Supplementary figure 6 | Multidirectional pencil beam architecture using a transmission diffraction grating.** Light enters the system using a 0.12 NA fiber-coupled laser and is collimated by lens L1 (*f* = 19 mm). The light is focused to a point by lens L2 (*f* = 50 mm) on a transmission diffraction grating, DG (80 grooves/mm, Dynasil). The diffracted light (*N* = 3 orders at -10, 0, and +10 deg.) is imaged by lens L3 (*f* = 75 mm) and relayed by lens L4 (*f* = 200 mm) onto a pivoting mirror located in a conjugate front focal plane of the illumination objective. Light is collected by a scan lens, L5 (*f* = 70 mm), and imaged onto a second scanning mirror positioned in a conjugate back focal plane of the illumination objective. The scanned light is imaged by a second scan lens, L6 (*f* = 70 mm), and relayed to the back focal plane of the objective by a tube lens, L7 (*f* = 165 mm). Finally, the light is focused into the cuvette and sample by the illumination objective (4x, NA = 0.28), resulting in an effective illumination NA*_x_* = 0.06 and NA*_y_* = 0.18. Only the scanning mirror is on for the mDSLM architecture using a transmission diffraction grating. The excited fluorescence is detected by a collection objective (16x, NA = 0.40), filtered by a bandpass filter, BP-F, and imaged onto a sCMOS camera using a tube lens, L8 (*f* = 100 mm).

**
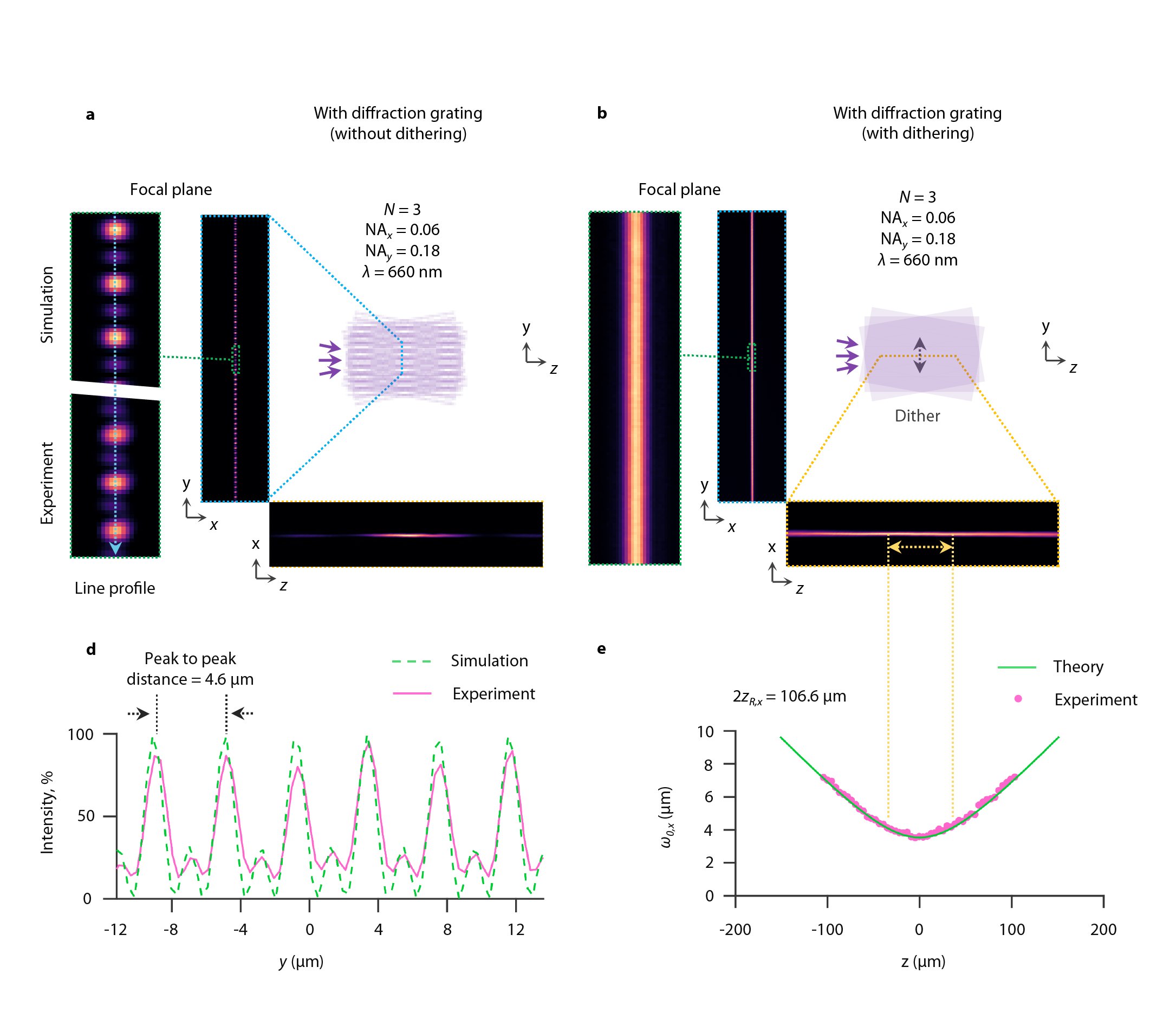
**

**Supplementary figure 7 | Simulations and experimental measurements of *N* = 3 overlapping light sheets.** In (a) the simulated and experimentally measured profiles of *N* = 3 overlapping 2D light sheets is shown. The overlapping light sheets are generated using a transmission diffraction grating (80 grooves/mm, Dynasil), where the 2D light sheets are oriented at angles of approximately -10, 0, and +10 deg. The corresponding intensity pattern after dithering of the beam in the *y*-direction is shown in (b). Images were recorded by reflecting the *xy* cross-section of the beams (*λ* = 660 nm) off of a flat mirror and scanning the mirror in the *z*-direction.

**
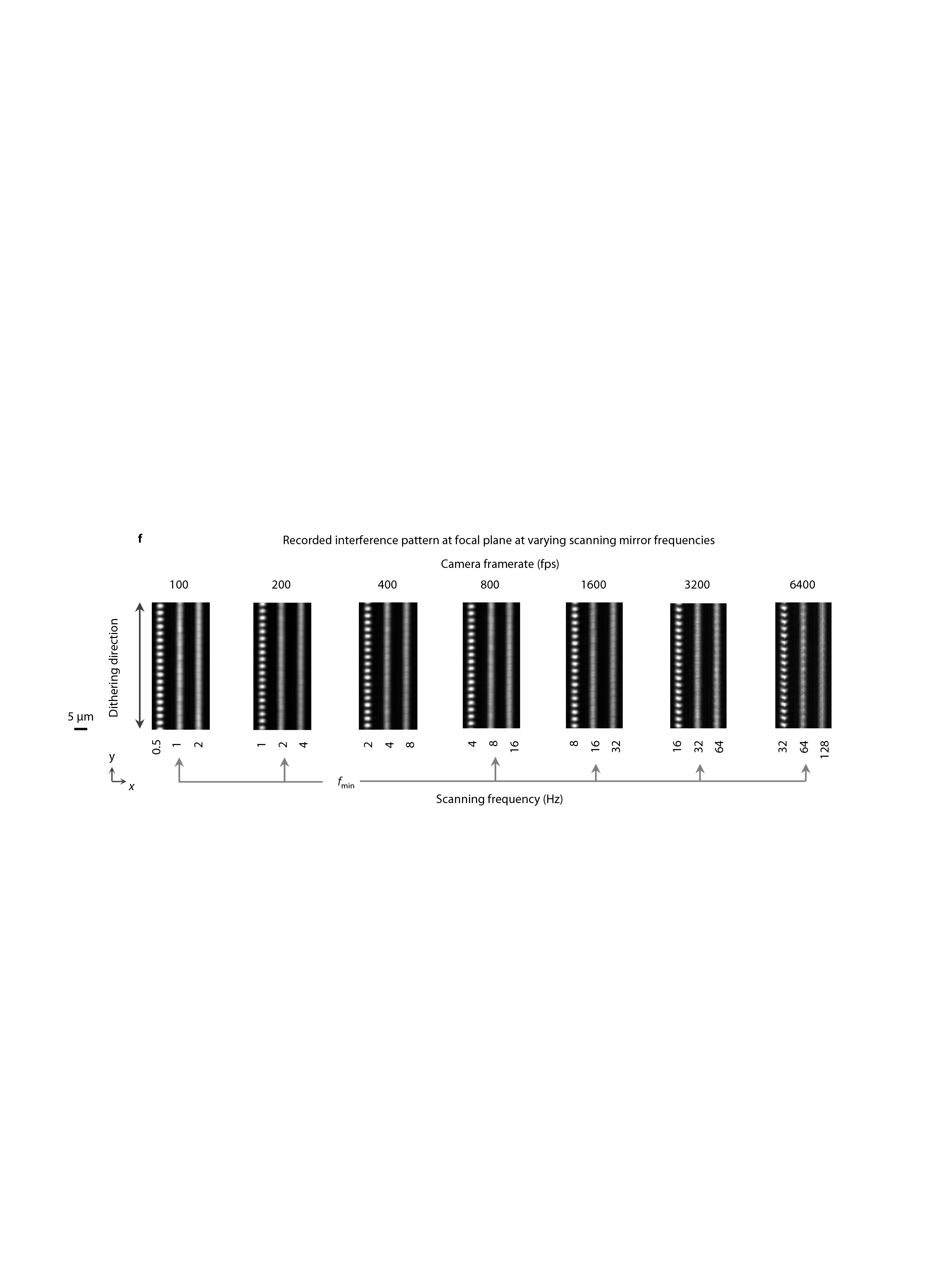
**

**Supplementary figure 8 | Experimental determination of the required scanning speed for dithering.** Images of the interference pattern generated by *N* = 3 overlapping 2D light sheets at the focal plane (*xy*-plane) are shown for various camera framerates and scanning mirror frequencies (*λ* = 660 nm). Unlike mSPIM, which requires a pivoting mirror to be actuated at a frequency greater than or equal to the camera framerate (to pivot a 2D light sheet at least once per camera exposure), the interference pattern generated by multiple overlapping sheets must only be translated by a distance greater than or equal to the distance between the peaks of the interference pattern. This is dependent on the number and angle of overlapping 2D light sheets and illumination optics, but in general results in a reduction in the speed requirement of the scanning mirror by several orders of magnitude. For example, for the illumination optics used in this study, and assuming a total scanning distance of 100 μm with the scanning mirror, the minimum required scanning frequency, *f_min_*, is two orders of magnitude less than the camera framerate.


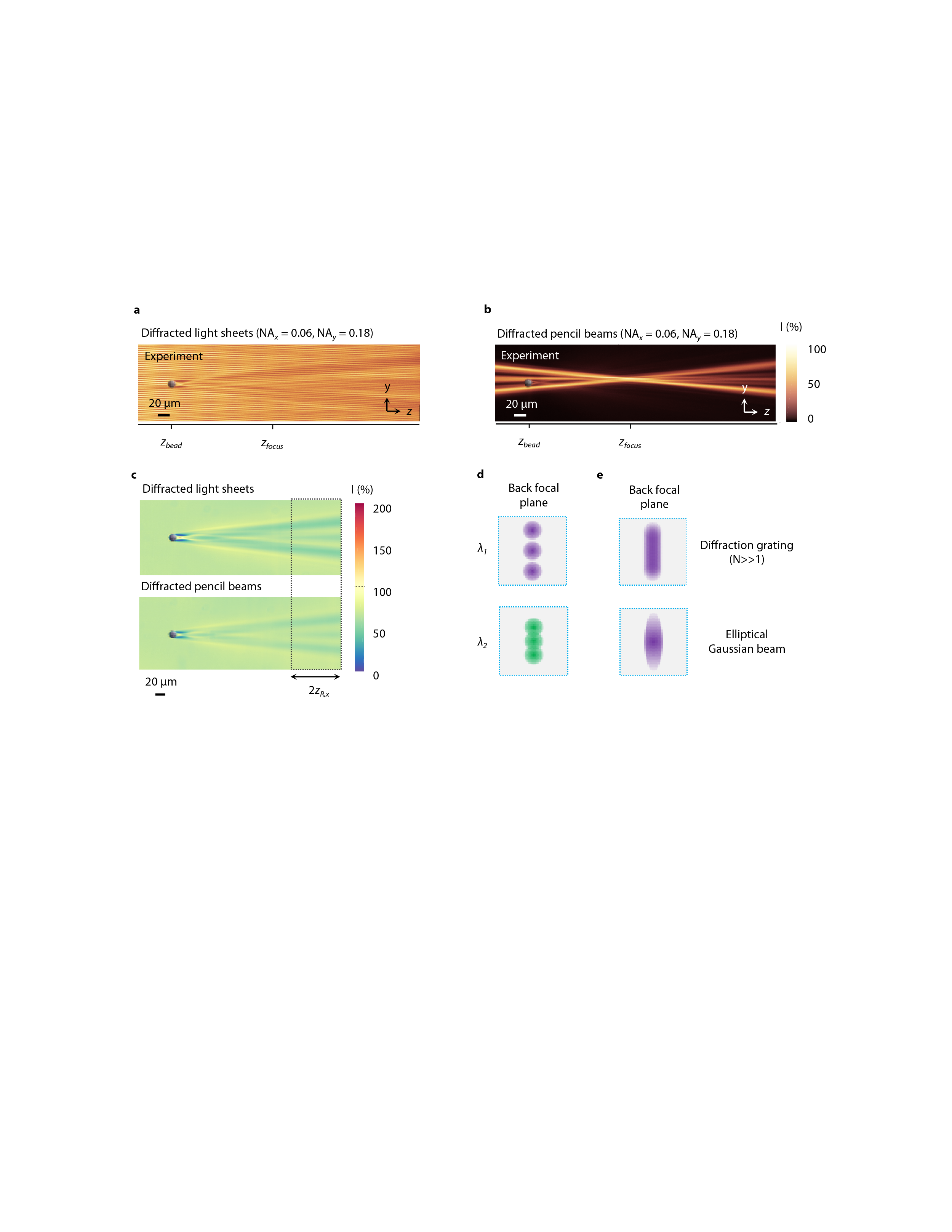


**Supplementary figure 9 | Experimental measurements of multidirectional illumination light sheet and pencil beams.**  In (a) an experimental image of the *N* = 3 diffracted light sheets propagating around a large glass sphere (*d* = 20 µm, *n_sphere_* = 1.59) in a fluorescent gel (*n_gel_* = 1.46) is shown (*λ* = 660 nm). The coherent Gaussian lattice pattern is visible, as well as three slight shadows corresponding to the three tilted light sheets. In (b) the corresponding experimental image of the diffracted Gaussian pencil beams are shown. Similar to mDSLM using an elliptical Gaussian beam, the three angled circular Gaussian beams are able to propagate around the glass sphere and reach the beam focus. The resulting images after scanning are shown in (c). Because a transmission diffraction grating is used, different wavelengths will be diffracted at different angles, and therefore at different positions along NA*_y_* in the back focal plane of the illumination objective. In addition, NA*_y_* is spanned by only a discrete number of diffraction orders, *N*. As *N* approaches infinity, the back focal plane resembles an elliptical Gaussian beam. A comparison of this case, relative to an elliptical Gaussian beam, is shown in (e).

**
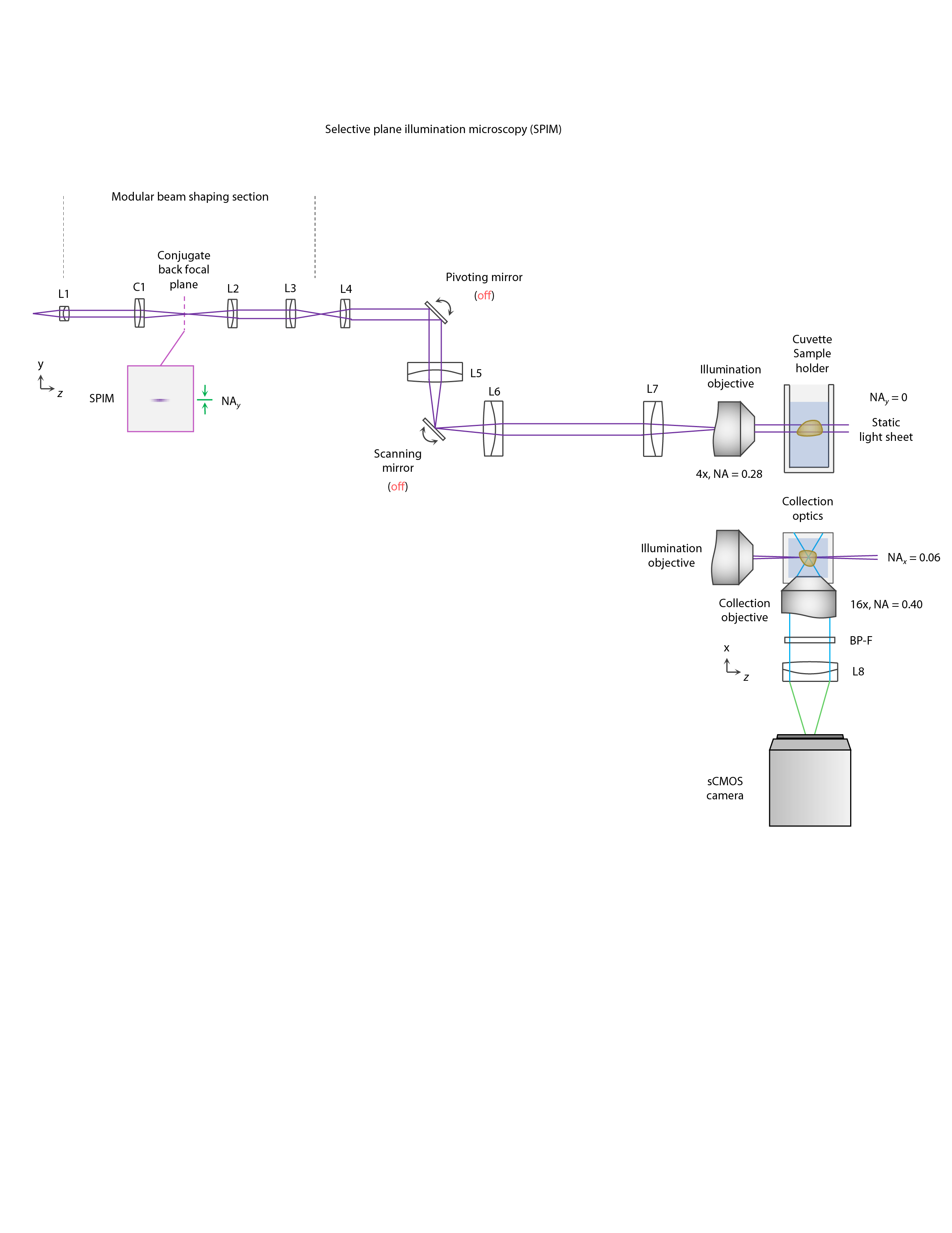
**

**Supplementary figure 10 | Experimental setup for the SPIM architecture.** Light enters the system using a 0.12 NA fiber-coupled laser and is collimated by lens L1 (*f* = 19 mm). The light is focused to a line by cylindrical lens C1 (*f* = 50 mm), and refocused by lenses L2 (*f* = 50 mm), L3 (*f* = 75 mm), and L4 (*f* = 200 mm) onto a pivoting mirror located in a conjugate front focal plane of the illumination objective. Light is collected by a scan lens, L5 (*f* = 70 mm), and imaged onto a second scanning mirror positioned in a conjugate back focal plane of the illumination objective. The scanned light is imaged by a second scan lens, L6 (*f* = 70 mm), and relayed to the back focal plane of the objective by a tube lens, L7 (*f* = 165 mm). Finally, the light is focused into the cuvette and sample by the illumination objective (4x, NA = 0.28), resulting in an effective illumination NA*_x_* = 0.06 and NA*_y_* = 0. Both the pivoting and scanning mirror are off for the SPIM architecture. The excited fluorescence is detected by a collection objective (16x, NA = 0.40), filtered by a bandpass filter, BP-F, and imaged onto a sCMOS camera using a tube lens, L8 (*f* = 100 mm).

**
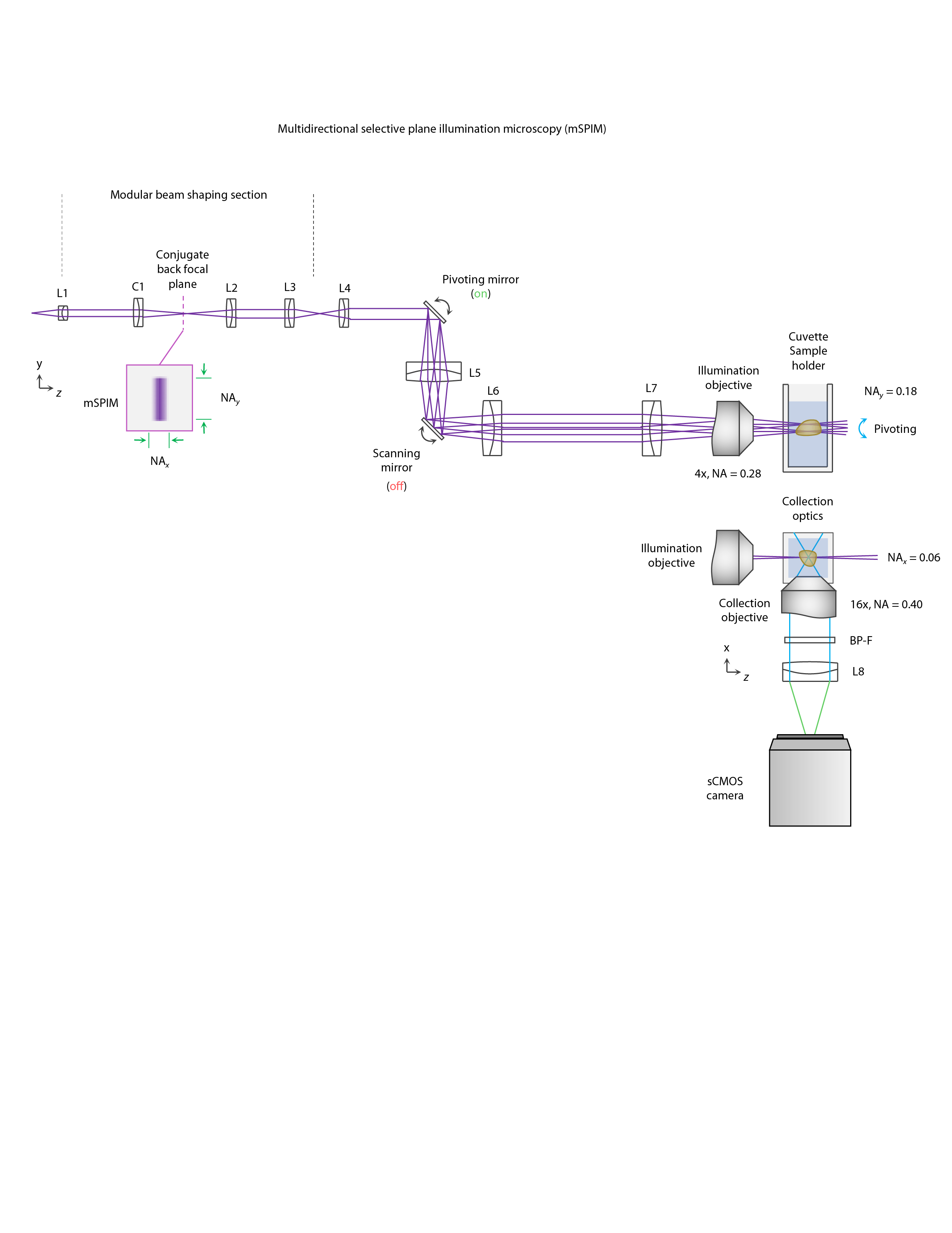
**

**Supplementary figure 11 | Experimental setup for the mSPIM architecture.**  Light enters the system using a 0.12 NA fiber-coupled laser and is collimated by lens L1 (*f* = 19 mm). The light is focused to a line by cylindrical lens C1 (*f* = 50 mm), and refocused by lenses L2 (*f* = 50 mm), L3 (*f* = 75 mm), and L4 (*f* = 200 mm) onto a pivoting mirror located in a conjugate front focal plane of the illumination objective. Light is collected by a scan lens, L5 (*f* = 70 mm), and imaged onto a second scanning mirror positioned in a conjugate back focal plane of the illumination objective. The scanned light is imaged by a second scan lens, L6 (*f* = 70 mm), and relayed to the back focal plane of the objective by a tube lens, L7 (*f* = 165 mm). Finally, the light is focused into the cuvette and sample by the illumination objective (4x, NA = 0.28), resulting in an effective illumination NA*_x_* = 0.06 and NA*_y_* = 0.18. For the mSPIM architecture, the pivoting mirror is on and pivots the beam within the sample by ~10 deg., resulting in NA*_y_* ~ 0.18. The excited fluorescence is detected by a collection objective (16x, NA = 0.40), filtered by a bandpass filter, BP-F, and imaged onto a sCMOS camera using a tube lens, L8 (*f* = 100 mm).

**
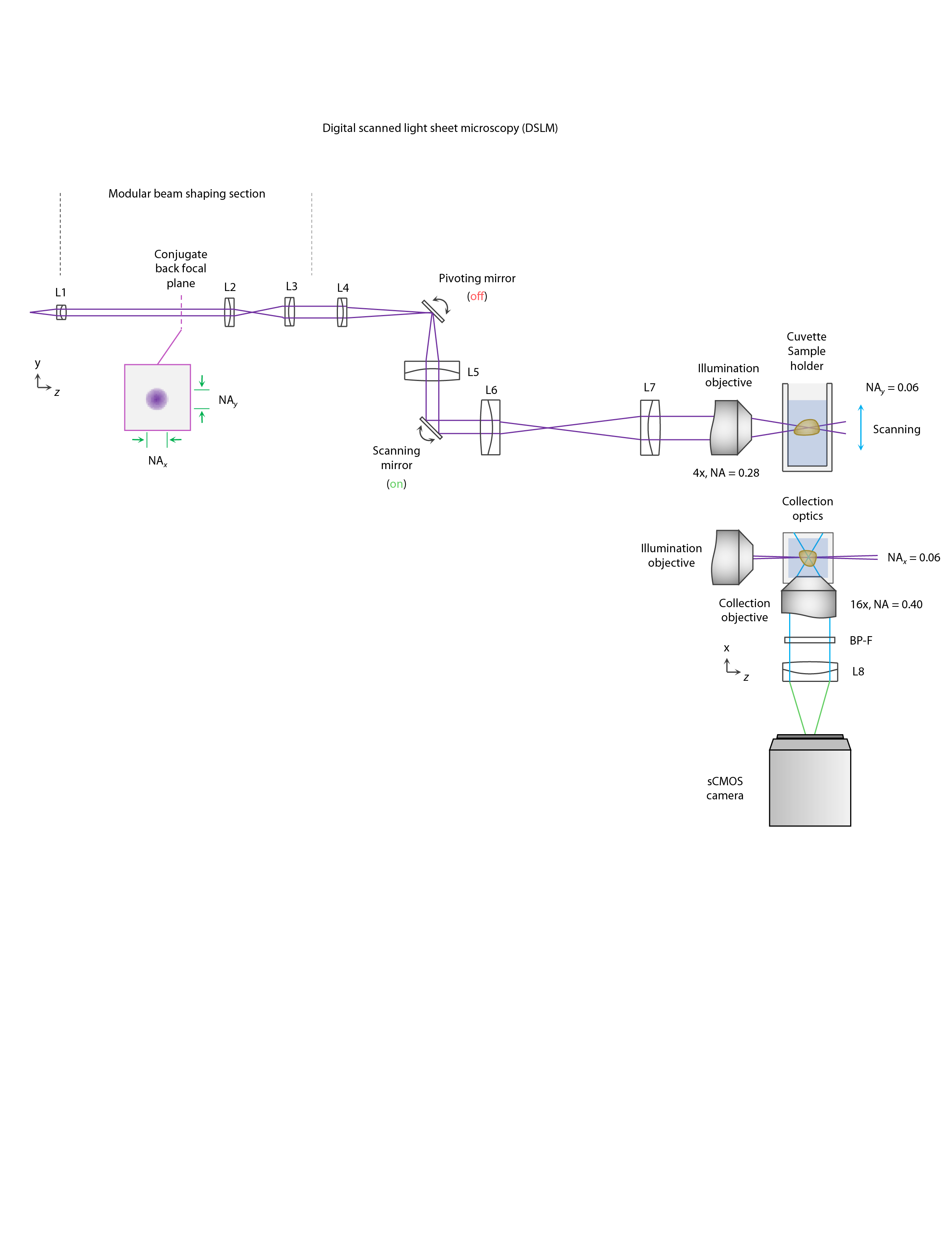
**

**Supplementary figure 12 | Experimental setup for the DSLM architecture.** Light enters the system using a 0.12 NA fiber-coupled laser and is collimated by lens L1 (*f* = 19 mm). The light is focused to a point by lens L2 (*f* = 50 mm) and imaged by lenses L3 (*f* = 75 mm) and L4 (*f* = 200 mm) onto a pivoting mirror located in a conjugate front focal plane of the illumination objective. Light is collected by a scan lens, L5 (*f* = 70 mm), and imaged onto a second scanning mirror positioned in a conjugate back focal plane of the illumination objective. The scanned light is imaged by a second scan lens, L6 (*f* = 70 mm), and relayed to the back focal plane of the objective by a tube lens, L7 (*f* = 165 mm). Finally, the light is focused into the cuvette and sample by the illumination objective (4x, NA = 0.28), resulting in an effective illumination NA*_x_* = 0.06 and NA*_y_* = 0.18. Only the scanning mirror is on for the DSLM architecture.

**
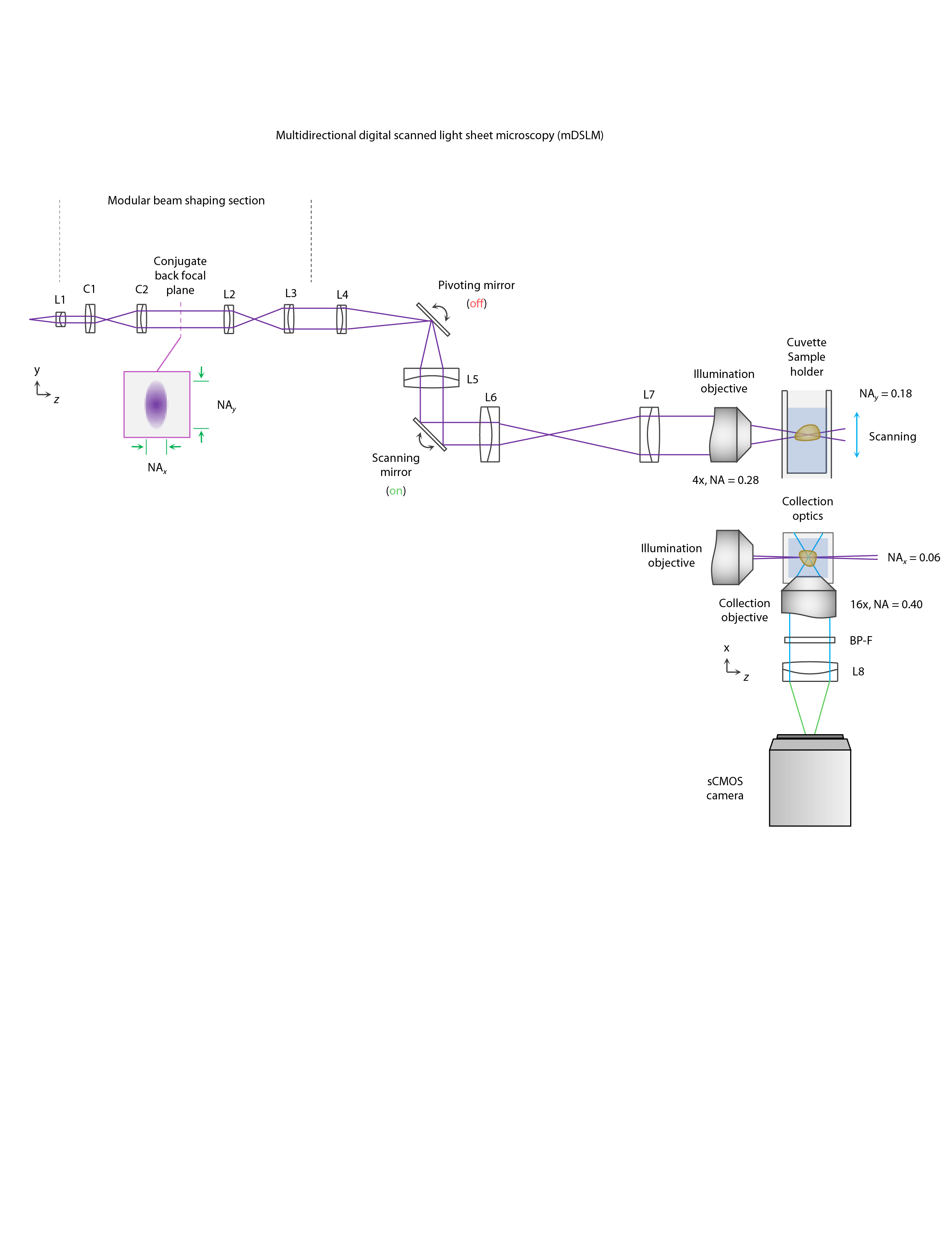
**

**Supplementary figure 15 | Experimental setup for the mDSLM architecture.** Light enters the system using a 0.12 NA fiber-coupled laser and is collimated by lens L1 (*f* = 19 mm). The light is expanded in the *y*-direction using a 3x cylindrical telescope, C1 (*f* = 50 mm) and C2 (*f* = 150 mm). The light is then imaged by lenses L2 (*f* = 50 mm), L3 (*f* = 75 mm), and L4 (*f* = 200 mm) onto a pivoting mirror located in a conjugate front focal plane of the illumination objective. Light is collected by a scan lens, L5 (*f* = 70 mm), and imaged onto a second scanning mirror positioned in a conjugate back focal plane of the illumination objective. The scanned light is imaged by a second scan lens, L6 (*f* = 70 mm), and relayed to the back focal plane of the objective by a tube lens, L7 (*f* = 165 mm). Finally, the light is focused into the cuvette and sample by the illumination objective (4x, NA = 0.28), resulting in an effective illumination NA*_x_* = 0.06 and NA*_y_* = 0.18. Only the scanning mirror is on for the mDSLM architecture.

**Supplementary video 1 | Circular and elliptical Gaussian beams propagating through a glass sphere.** Numerical simulations and experimentally measured images of circular (top) and elliptical (bottom) Gaussian beams propagating through a glass sphere. During scanning, the circular Gaussian beam is heavily refracted by the glass sphere, leading to a decrease in illumination intensity at the beam focus. This leads to an excitation shadowing artifact, which is mitigated by the elliptical Gaussian beam’s ability to propagate around the glass sphere.

**Supplementary video 2 | Comparison of SPIM, mSPIM, DSLM, and mDSLM imaging in biological tissue.** Video of *z*-stacks acquired in mouse small intestinal tissue using SPIM, mSPIM, DSLM, and mDSLM. With increasing depth, SPIM and mSPIM images show decreased contrast due to an increased collection of out-of-focus light. DSLM and mDSLM images show improved contrast due to the use of confocal line detection. At deeper depths the SPIM and DSLM images exhibit artifacts due to excitation shadowing. In contrast, the mSPIM and mDSLM images exhibit reduced shadowing artifacts due to multidirectional illumination.

**Supplementary video 3 | Video of *N* = 3 overlapping light sheets.** Numerical simulations and experimentally measured images of *N* = 3 overlapping light sheets generated by a diffraction grating propagating through a glass sphere are shown on top. On bottom, the cumulative image demonstrating the uniformity of an effective excitation beam after dithering is shown.

**Supplementary video 4 | Video of *N* = 3 overlapping pencil beams.** Numerical simulations and experimentally measured images of *N* = 3 overlapping pencil beams generated by a diffraction grating propagating through a glass sphere.
